# Dopamine beta-hydroxylase deficiency

**DOI:** 10.1186/1750-1172-1-7

**Published:** 2006-03-30

**Authors:** Jean-Michel Senard, Philippe Rouet

**Affiliations:** 1 Club d'Etude du Système Nerveux Autonome, Autonomic Unit of the Department of Clinical Pharmacology and INSERM U586, Faculté de Médecine, 37 allées Jules Guesde, 31073 Toulouse cedex, France; 2 INSERM Unit 586, Institut Louis Bugnard, C.H.U. Rangueil, 31054, Toulouse cedex, France

## Abstract

Dopamine beta-hydroxylase (DβH) deficiency is a very rare form of primary autonomic failure characterized by a complete absence of noradrenaline and adrenaline in plasma together with increased dopamine plasma levels. The prevalence of DβH deficiency is unknown. Only a limited number of cases with this disease have been reported. DβH deficiency is mainly characterized by cardiovascular disorders and severe orthostatic hypotension. First symptoms often start during a complicated perinatal period with hypotension, muscle hypotonia, hypothermia and hypoglycemia. Children with DβH deficiency exhibit reduced ability to exercise because of blood pressure inadaptation with exertion and syncope. Symptoms usually worsen progressively during late adolescence and early adulthood with severe orthostatic hypotension, eyelid ptosis, nasal stuffiness and sexual disorders. Limitation in standing tolerance, limited ability to exercise and traumatic morbidity related to falls and syncope may represent later evolution. The syndrome is caused by heterogeneous molecular alterations of the *DBH *gene and is inherited in an autosomal recessive manner. Restoration of plasma noradrenaline to the normal range can be achieved by therapy with the synthetic precursor of noradrenaline, L-threo-dihydroxyphenylserine (DOPS). Oral administration of 100 to 500 mg DOPS, twice or three times daily, increases blood pressure and reverses the orthostatic intolerance.

## Disease name and synonyms

Dopamine beta-hydroxylase deficiency

Norepinephrine deficiency

Noradrenaline deficiency

## Definition/diagnostic criteria

Dopamine beta-hydroxylase (DβH) deficiency is a very rare form of primary autonomic failure characterized by a complete absence of noradrenaline and adrenaline in plasma together with increased plasma dopamine levels. It was first described in 1986 [1]. This rare congenital disease is caused by a series of mutations in the *DBH *gene, mapped to chromosome 9q34, encoding the key enzyme in noradrenaline synthesis.

The diagnosis should be suspected when pure sympathetic autonomic failure is associated with absence of both noradrenaline and adrenaline and accumulation of dopamine in plasma.

## Epidemiology

The very limited number of cases of DβH deficiency in humans suggests that the disorder is very rare. However, since deficiency in DβH has been shown to be lethal in embryos or shortly after birth in mice [2], the prevalence of the disorder may be higher than expected. It should be kept in mind that history of spontaneous abortions and stillbirths has been noted in parents of DβH deficient patients. Among the cases reported, one family only contained two members presenting with the disease [3].

## Etiology

Despite the fact that the *DBH *gene was cloned a long time ago [4], the molecular defects in DβH deficiency are poorly understood. Access to extensive data concerning the *DBH *gene is available on the Internet [5]. In three unrelated patients, a common mutation has been identified in intron 1 (IVS1+2T→C) leading to aberrant splicing and a premature stop codon probably involved in noradrenaline deficiency [3,6]. In one patient, a mutation in exon 4 (764G>T) that leads to alteration of a sequence specific to copper type II ascorbate-dependent monooxygenase was also described [3]. A summary of identified mutations in the *DBH *gene is given in Table 1. However, these mutations cannot entirely explain the phenotype, since some of them are also found in healthy subjects [7]. It is possible, for instance, that the combination of the IVS1+2T→C mutation with other missense mutations is necessary for clinical expression of the disorder. Clearly, further studies are needed to explain how the identified mutations can lead to the absence of detectable enzyme protein [for a review see ref [8]].

**Table 1:** Summary of discovered mutations in *DBH *gene (extracted from ).

**Nucleotide change**	**Location**	**Amino acid change**	**Mutation type**
C-1021T	5' flanking		Noncoding
G259A	Exon 1	V87M	Missense
IVS1+2T→C	Intron 1		Noncoding
C300A	Exon 2	D100E	Missense
IVS3+8C→T	Intron 3		Noncoding
G991A	Exon 6	D331N	Missense
IVS10+415A0→G	Intron 10		Noncoding

From a pathophysiological point of view, whatever the nature of the mutations leading to DβH enzyme inactivity, the consequence in patients is a large accumulation of dopamine, the precursor of noradrenaline, in sympathetic nerves. This biochemical pattern probably explains cardiovascular paradoxical responses such as increase in dopamine but not in noradrenaline levels with standing or during hypoglycemia or tyramine infusion. It is, however, rather surprising, in view of the role of central nervous system DβH in mood and behavior [8,9], that only mild behavioral changes have been reported in DβH patients.

## Clinical description

The available data come from clinical descriptions of the reported cases (see Table 2). First symptoms often start during a complicated perinatal period with hypotension, muscle hypotonia, hypothermia and hypoglycemia. Delay in opening of the eyes, ptosis of eyelids and vomiting have also been reported. Children with DβH deficiency exhibit reduced ability to exercise because of blood pressure inadaptation with exertion and syncope. Symptoms usually progressively worsen during late adolescence and early adulthood with severe orthostatic hypotension, eyelid ptosis, nasal stuffiness and sexual disorders.

**Table 2:** Main clinical characteristics of DβH deficiency. Adapted from Robertson D *et al*., 1991 [13].

**Feature**	**(%)**
Severe orthostatic hypotension	100
Impaired ejaculation	100
Ptosis of eyelids	67
Complicated perinatal course	67
Nocturia	67
Hyperextensible/hyperflexible joints	50
High palate	50
Nasal stuffiness	50
Mild behavioral changes	33
Seizures (with hypotension)	33
Bradydactyly	33
Sluggish tendon reflexes	33
Weak facial musculature	33
Hypotonic skeletal muscles	33
Atrial fibrillation	16

Examination indicates low blood pressure in standing position, small but light- and accommodation-reactive pupils and normal sweating. Mild behavioral changes have also been reported in some patients. Some biochemical abnormalities have also been reported such as hypoprolactinemia, hypomagnesemia and raised blood urea nitrogen.

## Diagnostic methods

The clinical exploration of autonomic nervous system activity indicates a pure and isolated sympathetic failure with normal cholinergic function. Orthostatic hypotension is profound, and blood pressure does not increase as expected during usual tests such as Valsalva manoeuvre (phase IV), isometric handgrip or cold exposure. When pharmacological testing is performed, some responses clearly indicate adrenergic cardiac and vascular receptor supersensitivity. Drug challenge confirms the sympathetic failure with a lack of pressor response to tyramine or a significant and apparently paradoxical elevation of blood pressure with clonidine.

Since the key enzyme that catalyses the conversion of dopamine to noradrenaline, DβH, is undetectable in 4% of the population with normal concentrations of catecholamines [12], definite diagnosis of DβH deficiency is evident when plasma levels of catecholamines and metabolites are measured. In DβH deficient patients, circulating levels of noradrenaline and adrenaline are undetectable, whereas dopamine levels are elevated [13]. Metabolites of noradrenaline are absent in plasma, urine and cerebro-spinal fluid.

## Differential diagnosis

A typical flare reaction to intradermal histamine together with normal tearing sensory function, normal taste and smell, normal intact corneal and deep tendon reflexes, and lack of Askenazi Jewish extraction allow exclusion of familial dysautonomia [10]. Absence of profound mental retardation or hypopigmentation makes confusion with Menkes syndrome improbable [11].

## Genetic counseling

DβH deficiency is inherited in an autosomal recessive manner [6]. At conception, the sibs of an affected individual have a 25% chance of being affected, a 50% chance of being asymptomatic carriers, and a 25% chance of being unaffected and not carriers. The optimal time for determination of genetic risk is before pregnancy. *DBH *molecular genetic testing is available on a research basis only.

## Management including treatment

As orthostatic hypotension is the main symptom of patients with DβH deficiency, most of the available information on treatment focuses on this aspect. Many empirical therapies using mineralocorticoids or adrenergic receptor agonists have been reported to have mild effects. As the underlying biochemical defect has been identified, dihydroxyphenylserine (L-threo-3,4-dihydroxyphenylserine, L-Threo-DOPS, DOPS), a synthetic precursor of noradrenaline, is the treatment of choice. DOPS has been proposed for management of the orthostatic hypotension, with controversial results [14]. Administration of DOPS in mice lacking the *Dbh *gene restores plasma noradrenaline levels to normal and reverses behavioral abnormalities [15]. Restoration of the sensitivity to antidepressants has been demonstrated in the same model [9]. In humans with DβH deficiency, L-Threo-DOPS administration results in dramatic increase in blood pressure and relief of postural symptoms [16].

## Prognosis

Little is known about prognosis. Retrospective data indicate that in most described cases, the perinatal period is complicated by episodes of hypotension, hypoglycemia and hypothermia. Later evolution seems to be marked by progressive orthostatic hypotension with limitation in standing tolerance, limited ability to exercise and traumatic morbidity related to falls and syncope. L-threo-DOPS has been described as being very effective for restoring noradrenergic tone and correcting postural hypotension [1]. Effects of L-threo-DOPS on other aspects of autonomic failure have not been reported.
